# Association of Early Sports Practice with Cardiovascular Risk Factors in Community-Dwelling Adults: A Retrospective Epidemiological Study

**DOI:** 10.1186/s40798-023-00562-y

**Published:** 2023-02-21

**Authors:** Gabriela C. R. da Silva, William R. Tebar, Bruna T. C. Saraiva, Breno Q. Farah, Luiz Carlos M. Vanderlei, Gerson Ferrari, Diego Giulliano Destro Christofaro

**Affiliations:** 1 Department of Physical Education, School of Technology and Sciences, São Paulo State University (UNESP), Roberto Simonsen Street, No. 305, Presidente Prudente, São Paulo 19060-900 Brazil; 2 Universidade Federal Rural de Pernambuco (UFRP), Recife, Brazil; 3 Faculty of Health Sciences, Universidad Autónoma de Chile, 7500912 Providencia, Chile

**Keywords:** Physical activity, Cardiometabolic, Youth, Motor behavior, Health

## Abstract

**Background:**

Sports practice in childhood and adolescence has been inversely related to the chances of developing cardiovascular risk factors (CRFs). However, it is not clear whether sports practice in childhood and adolescence could be inversely related to CRF in adult life.

**Objectives:**

This study aimed to analyze the association between early sports practice and cardiovascular risk factors in a randomized sample of community-dwelling adults.

**Methods:**

For this, 265 adults aged ≥ 18 years composed the sample. Cardiovascular risk factors of obesity, central obesity, diabetes, dyslipidemia, and hypertension were collected. Early sports practice was retrospectively self-reported using an appropriate instrument. Total physical activity level was assessed by accelerometry. The association between early sports practice and cardiovascular risk factors in adulthood was analyzed by binary logistic regression, adjusted for sex, age, socioeconomic status, and moderate-to-vigorous physical activity.

**Results:**

Early sports practice was observed in 56.2% of the sample. The prevalence of central obesity (31.5 vs. 50.0%; *p* = 0.003), diabetes (4.7% vs. 13.7%; *p* = 0.014), dyslipidemia (10.7% vs. 24.1%; *p* = 0.005), and hypertension (14.1% vs. 34.5%; *p* = 0.001) was lower in participants who reported early sports practice. Participants who reported early sports practice in childhood and adolescence were, respectively, 60% (OR = 0.40; 95% CI 0.19–0.82) and 59% (OR = 0.41; 95% CI 0.21–0.82) less likely to have hypertension in adult life when compared to those with no early sports practice, independently of sex, age, socioeconomic status, and habitual physical activity level in adulthood.

**Conclusion:**

Early sports practice in childhood and adolescence was a protective factor for hypertension in adulthood.

## Key Points

- Sports practice has a protective effect against cardiovascular risk in children and adolescents.
- The prevalence of central obesity, diabetes, dyslipidemia and hypertension was lower in participants who reported early sports practice.
- Early sports practice was a protective factor against hypertension in adult life, independent of current physical activity level.

## Background

During youth, sports participation is an important practice of physical activity, which is a type of physical activity of higher intensities [1]. During this life stage, sports practice can occur both in the school environment (mainly in physical education classes) and also outside the school (in recreational leisure time in the community and clubs). In various countries, sports practice in childhood and adolescence is mainly linked to collective sports, such as soccer and volleyball [2].

Sports practice in childhood and adolescence has been considered a protective factor for a series of cardiovascular risk factors. In fact, a recent meta-analysis [3] showed that children and adolescents who participate in sports present better cardiovascular structure and function compared to those who do not participate, such as lower blood pressure values and carotid intimal thickness. In addition, original studies have shown that participation in sports is associated with better glucose values [4] and heart rate variability [5], dyslipidemias [6, 7], and cardiac autonomic modulation [8].

While research has shown early sport participation to influence the health of children and adolescents, select research has also demonstrated early sports participation to be inversely associated with cardiovascular risk factors in adult life. Fernandes et al. [9] in a study with more than 3000 adults observed that participants who played sports in childhood and adolescence were approximately 60% less likely to have dyslipidemia. Werneck et al. [10] observed that individuals who practiced sports in childhood and adolescence presented lower body adiposity, even if they were insufficiently active in adulthood.

Although the mechanisms behind these responses are still unknown, it is possible to speculate that early sports practice has a direct impact on the habitual level of physical activity in adult life. A systematic review study with 29 studies reported that early sports practice with a higher weekly frequency, persistence for at least 3 years of practice, and the highest level of practice/competition were associated with higher levels of physical activity in adulthood [11]. In this sense, it is well established in the literature that higher physical activity levels are associated with better cardiovascular outcomes [12–14].

Among the several benefits of physical activity to cardiovascular risk factors in adulthood, the potential role of early sports practice in improving adult physical activity level needs to be highlighted. Sports activities represent the most intense range of physical activity during youth [1], as they are organized activities with a planned schedule, duration, and weekly frequency [11]. Sports practice has been associated with better motor skills and physical fitness in youth [15–18], which can be carried into adulthood and enable greater adherence to the practice of physical activity in later periods of life. A recent study by Silva et al. [19] confirmed this hypothesis, reporting that early sports practice was associated with a higher level of vigorous physical activity in adulthood.

Despite these findings, there are still knowledge gaps in the current evidence in the literature. Studies that analyze the impact of early sports practice on adulthood health have failed to adjust their results for potential confounding factors, such as current physical activity level (mainly by objective measurement). Sex, age, and socioeconomic status are important confounding variables to be considered in the relationship between sports practice and its benefits [20, 21]. Moreover, engagement in early sports practice can vary between childhood and adolescence [22], and the possible benefits of sports practice on cardiovascular risk factors in adulthood still need to be better clarified according to each life stage. A systematic review reported that the majority of studies investigating early sports practice combine childhood and adolescence as only one life stage [11]. In this sense, a secondary objective of the present study was to separately analyze the early sports practice in childhood and adolescence to better understand the phenomenon. We hypothesized that early sports practice in adolescence may have a higher protective role in cardiovascular risk factors in adulthood than early sports practice in childhood, due to better practice memory. There is a long process of socialization that allows greater adherence to the practice of physical activity and a greater perception of health [23]. Nevertheless, our main hypothesis was that early sports practice, in at least childhood or adolescence, contributes to lower cardiovascular risk factors in adulthood when compared to individuals without any early sports practice.

Therefore, the aim of the present study was to verify whether early sports practice is associated with a lower prevalence of cardiovascular risk factors in adulthood and whether these possible associations are independent of the realization of physical activity at recommended levels (moderate-to-vigorous intensity) and sociodemographic factors in adulthood.

## Material and Methods

### Sample

The sample included adults aged ≥ 18 years, from the municipality of Santo Anastácio, located in the southeastern region of Brazil. Considering that the approximate population over 18 years of age in the city of Santo Anastácio is 16,000 inhabitants (IBGE), an odds ratio value of 0.35 [9] was adopted to calculate the sample size, with a power of 80% and an alpha of 5%, which determined a minimum sample size of 215 individuals. For the sample recruitment, we considered that the city of Santo Anastácio was divided into 35 census sectors, of which 25 are located in the urban region and were included in the sampling process. The number of inhabitants in each urban census sector of the city was obtained, and its proportionality was calculated in relation to the total population. This proportionality was applied for each census sector to acquire the minimum proportion of participants from the total sample size (i.e., urban sector 1 had 5.6% of the total population, so the minimum sample obtained in sector 1 needed to be 5.6% of the minimum total sample required: 12 subjects). Within each urban census sector, the squares, streets, and households were coded and randomly selected. The selected households were visited by the researchers, and all eligible subjects at home during the visit were invited to participate in the research. As many households as necessary to obtain the required sample were randomly selected. This methodology was previously published in the literature [24]. For the data collection, when the subject agreed to participate in the study, the accelerometer device was delivered to the home, with instructions given about its correct use, and a day was scheduled for the device to be collected and for administration of the face-to-face questionnaire.

As inclusion criteria, the participants were required to be 18 years of age or older, have resided in the city of Santo Anastácio for at least 2 years, sign the informed consent form agreeing to participate in the research, and answer the questionnaires. Exclusion criteria were inadequate accelerometer use and missing responses about the included variables on the questionnaire.

### Obesity, Central Obesity, Hypertension, Diabetes, and Dyslipidemia

Cardiovascular risk factors were composed of the absence or presence of general obesity (determined by body mass index), central obesity (determined by waist circumference measurement), diabetes, dyslipidemia (high triglycerides and/or high cholesterol), and arterial hypertension. Body mass was measured using a digital scale, with participants barefoot and wearing light clothes, while height was collected using a portable wall-fixed stadiometer. From these values, the body mass index was calculated by dividing the body mass by the height squared (BMI = kg/m^2^). Participants with a BMI ≥ 30 kg/m^2^ were classified as obese according to the World Health Organization [25]. Central fat was analyzed through waist circumference (in centimeters), collected at the middle point between the iliac crest and last rib, with an inextensible tape. Central obesity was defined as waist circumference > 102 for men and > 88 for women, according to cutoff points for metabolic risk [26].

The presence of cardiovascular risk factors (diabetes, dyslipidemia and hypertension) was assessed by two specific questions for each outcome: “*Do you currently have any of these problems, diagnosed by a doctor? Do you currently take any medication for these problems?*” Participants who answered “yes” to at least one of the two questions for each outcome were classified as having the cardiovascular risk factor.

### Early Sports Practice

Sports practice in childhood and adolescence was evaluated by the following questions: “When you were 7–10 years old, did you practice any supervised sports activity for at least one uninterrupted year outside of school (considering the vacation periods in the middle and end of the year)?”.

“When you were 11–17 years old, did you participate in any supervised sports activity for at least one uninterrupted school year (considering the vacation periods in the middle and end of the year)?” School physical education classes were excluded, but participation in school team training was considered. The possible answers to each question were “yes” or “no” [9, 27, 28].

For the purposes of the present study, supervised sports activities were those related to traditional sports modalities, practiced as an individual or in a team: soccer, volleyball, basketball, handball, tennis, swimming, gymnastic, athletics, judo, karate, and muay thai. Because the prevalence of sports practice may have been different in childhood and adolescence, we chose to evaluate this information separately in the current study.

### Moderate-to-Vigorous Physical Activity (MVPA)

MVPA was assessed using the GT3-X accelerometer (ActiGraph, LLC, Pensacola, FL, USA). For data cleaning, the ActiLife 6 program (ActiGraph, LLC, Pensacola, FL, USA) was used. Participants were instructed to wear the accelerometer for a period of 7 days, where a minimum of 5 days (including ≥ 1 weekend day) with ten full hours was considered as the minimum time to enter the data analysis. Each data sample, determined by counts, was summarized considering a determined interval of time, designated an epoch, which had a duration of 60 s. The period of 60 s was selected because it is the closest to the pattern of low-intensity and long-duration activity, allowing the physical activity to be counted in minutes per week [29]. Consecutive hours of zero counts and days with less than 10 h of monitoring were excluded [30]. Participants were instructed to remove the accelerometer only during waking hours and when bathing or in water activities. The cutoff proposed by Sasaki et al. [31] was adopted to identify the MVPA, where the accelerometer-registered activities with ≥ 2690 counts per minute were counted as moderate-to-vigorous intensity, resulting in the weekly level of physical activity for each participant (minutes per week in MVPA).

### Socioeconomic Status

The socioeconomic status was assessed by Brazilian Criteria for Economic Classification [32], which considers the educational level of participants, the presence and quantity of specific rooms and consumer goods at home, in addition to the urban infrastructure of the household and the presence of housemaids. From this information, a score is generated that the higher the value obtained, the better the socioeconomic status of the participant.

### Statistical Analysis

Descriptive statistics included mean, standard deviation, frequency, and proportions of early sports practice. The comparisons between the continuous variables in the characterization of the sample according to the periods of physical activity practice in childhood and/or adolescence were performed using the *t* test for independent samples. The comparisons of the frequencies of cardiovascular risk factors according to the periods of sports practice were performed using the chi-square test.

The association between cardiovascular risk factors and previous sports practice (practice in childhood and adolescence) was evaluated using binary logistic regression (OR, confidence interval 95%). For this, three models were created (model 1: crude; model 2: adjusted for sex, age, and socioeconomic level; model 3: model 2 + adjusted MVPA assessed by accelerometer). In order to verify the associations between the different stages of life, the results were presented stratified considering the practice of sports in childhood and adolescence. Effect sizes for significant associations were calculated by squaring the correlation value between the outcome and predicted probability value obtained from logistic regression models, with *r*-values classified as small (< 0.1), moderate (around 0.3), or strong (> 0.5), according to the benchmarks suggested by Cohen [33]. The adopted statistical significance was 5% and the confidence interval was 95%. The statistical package used was SPSS 15.0 (SPSS Inc., IBM Corp., Armonk, New York, NY, USA).

## Results

The sample of the present study consisted of 265 participants with a mean age of 42.3 (± 17.0) years. Early sports practice was observed in 56.2% of the sample (*n* = 149), where 42.6% of the sample (*n* = 113) reported sports practice in childhood and 49.1% (*n* = 130) reported sports practice in adolescence. Table 1 presents the variables for characterizing the sample according to sports practice in childhood and adolescence.

**Table 1:** Sample characterization according to early sports practice (*n* = 265)

	No early sports practice (*n* = 116)	Early sports practice (*n* = 149)	*p*
Age (years), mean (SD)	50.3 (16.5)	36.4 (14.9)	< 0.001
Body weight (kg), mean (SD)	73.9 (14.1)	79.6 (16.8)	0.005
Height (cm), mean (SD)	1.60 (0.09)	1.69 (0.08)	< 0.001
Waist circumference (cm), mean (SD)	92.1 (14.1)	89.0 (14.3)	0.236
Body mass index (kg/m^2^), mean (SD)	28.8 (5.2)	27.5 (5.2)	0.076
MVPA (minutes/week)	135.1 (178.7)	165.0 (154.2)	0.048
Socioeconomic status (ABEP score)	30.6 (8.6)	34.6 (9.1)	< 0.001
Sex			
Men, *n* (%)	24 (20.7)	89 (59.7)	< 0.001
Women, *n* (%)	92 (79.3)	60 (40.3)	
Obesity, *n* (%)	45 (38.7)	44 (29.5)	0.131
Central obesity, *n* (%)	58 (50.0)	47 (31.5)	0.003
Dyslipidemia, *n* (%)	28 (24.1)	16 (10.7)	0.005
Diabetes, *n* (%)	16 (13.7)	7 (4.7)	0.014
Hypertension, *n* (%)	40 (34.5)	21 (14.1)	< 0.001

*SD* standard deviation, *IR* interquartile range, *MVPA* moderate-to-vigorous physical activity

**Fig. 1:**
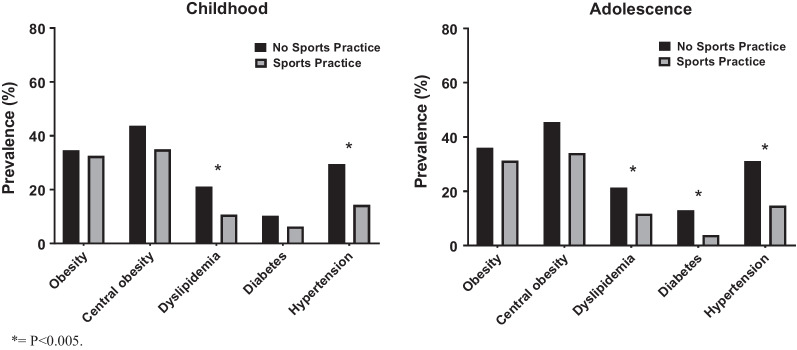
Prevalence of cardiovascular risk factors in adulthood according to sports practice in childhood and adolescence. *p < 0.005

Figure 1 presents information on the prevalence of cardiovascular risk factors in adulthood according to sports practice in childhood and adolescence. Participants with sports practice only in childhood showed a lower prevalence of dyslipidemia (10.7% vs. 21.1%, *p* = 0.026) and hypertension (14.3% vs. 29.5%, *p* = 0.004) than those without sports practice. Participants with sports practice only in adolescence showed a lower prevalence of dyslipidemia (11.7% vs. 21.4%, *p* = 0.037), diabetes (3.9% vs. 13.0%, *p* = 0.009), and hypertension (14.7% vs. 31.1%, *p* = 0.002) when compared to those without sports practice in this life stage.

The association between sports in childhood and cardiovascular risk factors is given in Table 2. Participants who were active in childhood in relation to sports were, respectively, 55% and 61% less likely to have dyslipidemia (odds ratio 0.45 [95% confidence interval: 0.22; 0.92], *p* = 0.029) and hypertension (odds ratio 0.39 [95% confidence interval: 0.21; 0.75], *p* = 0.004) in adulthood, considering the crude model. However, the association between childhood sports practice and dyslipidemia was attenuated and not statistically significant after adjustment for confounding factors, including MVPA in the adjustments. The association between childhood sports practice, on the other hand, remained associated with hypertension, even after adjusting for confounding factors (odds ratio 0.40 [95% confidence interval: 0.19; 0.82], *p* = 0.013). However, all the significant associations presented a small effect size (lower than 0.20).

**Table 2:** Association between early sports practice in childhood and cardiovascular risk factors in adulthood (*n* = 265)

	Sports practice in childhood			Effect size
	OR	95% CI	*p* value	
*Obesity*				
Model 1	0.91	0.51–1.63	0.754	–
Model 2	1.09	0.55–2.19	0.802	–
Model 3	1.09	0.54–2.18	0.812	–
*Central obesity*				
Model 1	0.69	0.41–1.14	0.152	–
Model 2	0.95	0.53–1.70	0.864	–
Model 3	0.91	0.51–1.63	0.752	–
*Dyslipidemia*				
Model 1	**0.45**	**0.22–0.92**	**0.029**	**0.02 (small)**
Model 2	1.06	0.46–2.45	0.888	–
Model 3	1.08	0.46–2.53	0.867	–
*Diabetes*				
Model 1	0.58	0.23–1.49	0.263	–
Model 2	1.34	0.43–4.13	0.615	–
Model 3	1.21	0.39–3.77	0.748	–
*Hypertension*				
Model 1	**0.39**	**0.21–0.75**	**0.004**	**0.03 (small)**
Model 2	**0.42**	**0.20–0.86**	**0.018**	**0.07 (small)**
Model 3	**0.40**	**0.19–0.82**	**0.013**	**0.07 (small)**

*OR* odds ratio, *CI* confidence interval, *Model 1* unadjusted, *Model 2* adjusted for sex, age, and socioeconomic status, *Model 3* variables of Model 2 plus weekly amount of moderate-to-vigorous physical activity

Bold: Statistically significant

Sports practice in adolescence was inversely associated with dyslipidemia (odds ratio 0.49 [95% confidence interval: 0.25; 0.97], *p* = 0.039), diabetes (odds ratio 0.27 [95% confidence interval: 0.09; 0.76], *p* = 0.013), and hypertension (odds ratio 0.38 [95% confidence interval: 0.20; 0.70], *p* = 0.002). After the insertion of the adjustment variables, there was attenuation and no longer statistically significant between the practice of sports in adolescence and diabetes and dyslipidemia. (This relationship remained nonsignificant even after the insertion of MVPA.) Alternatively, participants who reported early sports practice in adolescence were 59% less likely to have hypertension in adulthood, even after adjustments made in the multivariate model (odds ratio 0.41 [95% confidence interval: 0.21; 0.82], *p* = 0.013). However, as observed with early sports practice in childhood, all the significant associations presented a small effect size (lower than 0.20) (Table 3).

**Table 3:** Association between early sports practice in adolescence and cardiovascular risk factors in adulthood (*n* = 265)

	Sports practice in adolescence			Effect size
	OR	95% CI	*p* value	
*Obesity*				
Model 1	0.81	0.46–1.44	0.474	–
Model 2	0.94	0.49–1.80	0.845	–
Model 3	0.94	0.49–1.81	0.850	–
*Central obesity*				
Model 1	0.62	0.37–1.02	0.062	–
Model 2	0.81	0.47–1.42	0.476	–
Model 3	0.81	0.46–1.41	0.450	–
*Dyslipidemia*				
Model 1	**0.49**	**0.25–0.97**	**0.039**	**0.02 (small)**
Model 2	1.04	0.47–2.31	0.915	–
Model 3	1.04	0.47–2.31	0.918	–
*Diabetes*				
Model 1	**0.27**	**0.09–0.76**	**0.013**	**0.03 (small)**
Model 2	0.44	0.14–1.40	0.167	–
Model 3	0.43	0.13–1.36	0.149	–
*Hypertension*				
Model 1	**0.38**	**0.20–0.70**	**0.002**	**0.04 (small)**
Model 2	**0.41**	**0.21–0.82**	**0.012**	**0.07 (small)**
Model 3	**0.41**	**0.21–0.82**	**0.013**	**0.08 (small)**

*OR* odds ratio, *CI* confidence interval, *Model 1* unadjusted, *Model 2* adjusted for sex, age, and socioeconomic status, *Model 3* variables of Model 2 plus weekly amount of moderate-to-vigorous physical activity

Bold: Statistically significant

## Discussion

Among the main findings of the present study, it was observed that sports practice in childhood and adolescence was related to lower chances of presenting dyslipidemia in adulthood, and only sports practice performed in adolescence was related to lower chances of diabetes in adulthood, but in both cases, statistical significance was lost after adjustment for confounding variables, including moderate/vigorous physical activity in adulthood. However, both sports practice in childhood and in adolescence were considered protective factors for hypertension, independently of sex, age, socioeconomic status, and habitual physical activity in adult life.

These results corroborate in part with those observed by Fernandes et al. [9] who found that adults who reported physical activity in childhood and adolescence were less likely to present obesity and dyslipidemia during adulthood. However, the association of early sports practice with dyslipidemia and diabetes in the present study lost significance in the analysis adjusted by sex, age, and socioeconomic status (model 2) and with the addition of current physical activity level (model 3). One of the mechanisms hypothesized for the inverse association between early sports practice and obesity is that previous sports practice could contribute to the maintenance of body weight and, as a consequence, adiposity. This factor could contribute to the reduction in some adipokines released in inflammatory processes, such as interleukin 6 and tumor necrosis factor-alpha [34]. In the study by Fernandes et al. [9], these results remained even in the adjusted analyses.

The covariates of sex, age, and socioeconomic status were important confounders in the associations of the present study. Regarding age, it is possible that cardiovascular risk factors were more prevalent in older participants as pointed out by Rodgers et al. [35], it being observed that participants without early sports participation were older than those with early sports participation (50.3 vs. 36.4 years of age). With respect to sex, a higher prevalence of early sports participation was observed in men than in women in the present study (77.4 vs. 39.0). In addition, men were more likely to present cardiovascular risk factors when compared to women [36]. In relation to socioeconomic status, it has been reported that a better economic condition is associated with lower cardiovascular risk factors [37] and higher physical activity level in leisure time [38]. A higher socioeconomic level was also associated with higher sport participation in youth [39].

Studies in the literature show that MVPA is able to improve metabolic parameters such as dyslipidemia and diabetes. In a cohort study with approximately 10,000 participants, Yerramalla et al. [40] observed that the practice of moderate/vigorous physical activity was a protective factor for the development of diabetes and all-cause mortality in the most active subjects. Similar findings were also observed by Nagata et al. [41] in a cohort study over 30 years, considering metabolic variables, such as cholesterol and triglycerides. One of the possible reasons is that MVPA could contribute to improving reverse cholesterol transport and to accelerating increases in high-density lipoprotein cholesterol levels [42] and could also help in glycemic control [43]. For these aforementioned reasons, the association of early sports practice with dyslipidemia and diabetes in the present study may have been mitigated when controlling for MVPA. One of the advances in our study when compared to the studies by Yerramalla et al. [40] and Nagata et al. [41] was that we performed the MVPA assessment through accelerometry, which provides more accurate information regarding the intensity of physical activity than a questionnaire [44], minimizing recall bias and misclassification of physical activity intensities. A large cohort study [45] observed a constant reduction in cardiovascular and mortality risk, with higher volumes of moderate-to-vigorous intensity physical activity, reinforcing the importance of investigating physical activity determinants in adulthood, as in the case of early sports practice.

Participants with sports practice in childhood and adolescence were 60% and 59% less likely to present hypertension in adulthood, respectively. One of the possible reasons for these results is that physical activity in childhood and adolescence could reduce inflammatory parameters such as C-reactive protein [10], which would be linked to lower sympathetic activity and successively lower chances of having hypertension. Another hypothesis relates to the effects that previous sports practice could have on the vascular system throughout life, as well as improving cardiac autonomic modulation [46]. Werneck et al. [47] in a study with 107 adults, observed that adults who practiced physical activity in childhood and adolescence had lower carotid thickness when compared to their inactive peers at these stages of life. Leisure-time physical activity throughout life could be linked to greater elasticity of the carotid artery in adulthood [48], which would contribute to the better arterial flow and successively lower chances of arterial hypertension, and sport is the most widely practiced form of leisure-time physical activity in children and adolescents worldwide [1].

Another possible hypothesis for the inverse association between early sports practice and hypertension is that the practice of sports provides health improvements which can be tracked along the life stages. A recent systematic review reported that sports participation promotes benefits to cardiovascular structure and function in adolescence [3]*.* In addition, sports are the most intense physical activities practiced during this life stage [1], being associated with better physical fitness [15–18], which is an important factor for cardiovascular health and prevention of hypertension [45]. However, we do not know whether the participants in the present study practiced other physical activities during youth besides sports, which limits further comparisons in other contexts.

The extrapolation of the findings of the present study is importantly limited by residual confounding, since other important traditional cardiovascular risk factors were not assessed, such as smoking status, and a family history of heart disease. Zhou et al. [49] reported that smoking was associated with an increase in blood pressure, but not highly associated with the risk of hypertension development. Kaplan et al. [50] observed that current smoking raises the risk of hypertension by 30%. In addition, Chacko et al. [51] reported that a family history of cardiovascular disease is an independent risk factor for premature cardiovascular heart disease. It is possible that traditional cardiovascular risk factors could be interrelated, increasing cardiovascular risk beyond protective factors, such as physical activity.

Another important aspect to highlight is that participants who reported early sports practice were significantly younger than those who reported not having early sports practice (36.4 vs. 50.3 years of age). In addition, it has been reported that self-reported sports participation has been increasing for many decades [52], and, in this sense, it is possible that younger adults were more likely to have practiced sports during their youth than older adults.

Nevertheless, the higher age of participants with non-sports practice during youth could have substantially contributed to the worsening of cardiovascular risk factors in the present study. The aging process is related to oxidative stress, low-grade inflammation, and detrimental alterations in body cells [53] and is associated with arterial stiffness, lumen dilation, and wall thickening, leading to arterial damage [54]. In addition, the presence of atherosclerotic plaque in arterial walls causes the artery to narrow [55]. These factors are strongly associated with hypertension [56, 57]. Impaired renal function, cardiac autonomic modulation, and dysfunctions in glucose metabolism, leading to impairment in glycemic control and increased insulin resistance, could also contribute to an increase in hypertension rates during the aging process [58–60]. Furthermore, age-related disorders in lipoprotein metabolism, such as the liver sinusoidal endothelium, postprandial lipemia, an increase in free fatty acids, and deficiency in growth hormone also contribute to a higher risk of dyslipidemia in older participants [61]. In this sense, the significant difference in the age of participants who reported early sports practice, when compared to those who did not, could explain the loss of significance in our statistical model adjusted by sociodemographic factors (Model 2).

We can mention the lack of information on preexisting diseases in childhood and adolescence and on the weekly frequency and type of sports practice practiced during childhood and/or adolescence, which could influence the results observed. Other types of lifestyle habits, such as diet, sedentary behavior, and occupational demands developed throughout life that may influence cardiovascular health, but were not evaluated in the present study, should be considered. Another limitation to be considered is the self-reported medical diagnosis and medication use for cardiovascular risk factors, which precluded investigation of the disease burden. Studies with larger samples are recommended to enable sensitivity analysis according to different groups of age, sex, and socioeconomic status, which showed a substantial confounding role in our findings. Studies addressing comparisons between early sports practice vs. non-sport physical activity during youth are also encouraged to investigate the different effects on cardiovascular risk at later life stages.

Otherwise, as positive aspects, we emphasize the random sample selection process, providing an opportunity for all residents of the city where the study was carried out to participate in the study. Another positive point was the adjustment for the habitual physical activity level, measured objectively, which, in addition to considering possible acute effects of current physical activity, minimizes biases in memory and in the intensity classification of the participants.

## Conclusions

In summary, the present study observed that early sports practice was inversely associated with hypertension in adult life, even after adjustment for sex, age, socioeconomic status, and MVPA. It is suggested that health promotion actions against cardiovascular risk factors encourage the practice of sports from early ages.

## Data Availability

Data from this study may be available from the corresponding author upon reasonable request.
